# High-dose medium-term HMB supplementation did not trigger body composition changes in trained and untrained males under usual conditions or high-intensity functional exercise

**DOI:** 10.3389/fnut.2025.1681465

**Published:** 2025-09-24

**Authors:** Krzysztof Durkalec-Michalski, Magdalena Czlapka-Matyasik, Tomasz Podgórski, Małgorzata Marchelek-Myśliwiec, Paulina M. Nowaczyk

**Affiliations:** ^1^Department of Sports Dietetics, Poznan University of Physical Education, Poznań, Poland; ^2^Polish Society of Nutritional Sciences, Warsaw, Poland; ^3^Sport Sciences–Biomedical Department, Faculty of Physical Education and Sport, Charles University, Prague, Czechia; ^4^Department of Human Nutrition and Dietetics, Poznań University of Life Sciences, Poznań, Poland; ^5^Department of Biochemistry, Poznan University of Physical Education, Poznań, Poland; ^6^Department of Nephrology, Transplantology and Internal Medicine, Pomeranian Medical University, Szczecin, Poland

**Keywords:** beta-hydroxy beta-methyl butyrate acid, ergogenic support, nutrition, sports dietetics, personalized supplementation

## Abstract

**Introduction:**

*β*-hydroxy-β-methylbutyrate (HMB) supplementation may support fat-free mass (FFM) increase and fat mass (FM) decrease. Its utility has been studied mainly for 3 g_HMB_·day^−1^ and long-term supplementation (e.g., 12 weeks). Therefore, new and personalized effective HMB supplementation protocols should be verified.

**Methods:**

Ninety trained (TR, *n* = 53; 29.1 ± 7.7 years; FFM: 84.1 ± 5.1%) and untrained (UTR, *n* = 37; 32.3 ± 7.6 years; FFM: 75.7 ± 7.7%) males completed the randomized parallel-group placebo (PLA)-controlled study aiming at evaluating the influence of new individualized high-dose mid-term liquid HMB free acid supplementation protocol (90 mg_HMB_·kg_FFM_^−1^·day^−1^) alone (3 weeks; first period) and combined with high-intensity functional training (HIFT; 3 weeks; second period) on body mass (BM), FFM and FM, and total body water (TBW). The *Fight Gone Bad* (FGB) workout was an additional HIFT stimuli (2 units·week^−1^). Testing was performed at baseline (*BAS*) and after the first *(SUP*) and the second *(SUP+FGB*) study periods.

**Results:**

HMB doses were 4.8–7.8 g_HMB_·day^−1^. The intervention had no significant effect on BM, FFM, FM, or TBW. BM, FFM (kg), TBW, and TBW/FFM were higher at *SUP+FGB* vs. *BAS* regardless of the implemented treatment and training status. Nevertheless, there was an impact (*p* < 0.05) from training status (but not HMB/PLA) on FM (kg; slight increases in UTR) and TBW (slight decreases in UTR).

**Discussion:**

The individually adjusted high HMB dose did not change body mass and composition in trained or untrained individuals during a three-week exclusive supplementation or three-week supplementation in combination with additional HIFT stimuli. Therefore, any modifications in this area may likely require a longer treatment period.

## 1 Introduction

*β*-hydroxy-β-methylbutyrate (HMB) is a biologically active metabolite formed from the essential branched-chain amino acid leucine (Leu) through its intermediate, *α*-ketoisocaproic acid (1–5). In humans, only approximately 5% of Leu is converted into HMB under physiological conditions, resulting in a modest endogenous production of about 0.2–0.4 g_HMB_·day^−1^ (1, 3, 5). Given its limited synthesis and the low natural abundance of HMB in conventional food sources, achieving the most commonly studied dose of 3 g_HMB_·day^−1^ requires targeted dietary supplementation (1, 3).

Changes in muscle mass are regulated by the balance of muscle protein synthesis (MPS) and muscle protein breakdown (MPB) (6). HMB supports skeletal muscle maintenance and hypertrophy primarily *via* two complementary mechanisms: (1) stimulation of MPS through the activation of anabolic signaling pathways, including upregulation of the mammalian target of rapamycin (mTOR), and influence on its downstream targets ribosomal protein S6 kinase beta-1 (p70S6K1; activated through phosphorylation by mTORC1) and eukaryotic initiation factor-4 binding protein-1 (4E-BP1; phosphorylation by mTORC1 inactivates 4E-BP1, allowing translation to proceed) (7, 8); and (2) reduction of MPB *via* the inhibition of catabolic systems such as the ubiquitin–proteasome pathway and caspase activity, particularly under catabolic conditions (8) (e.g., illness or injury, prolonged inactivity or immobilization, sarcopenia, severe energy or protein restriction, or intense or prolonged physical exercise without adequate recovery). Moreover, recent preliminary studies by Duan et al. (9) on the 3 T3-L1 mice cell line also suggest that HMB increases both basal and maximal mitochondrial respiration, ATP production, and mitochondrial membrane permeability, which translates into enhanced fatty acid oxidation. This improved mitochondrial function in a broader context may contribute to changes in body composition by promoting a reduction in fat mass. Despite these proposed mechanisms by which HMB may influence MPS/MPB and fat utilization, the results of studies evaluating the effects of HMB supplementation on body mass and composition in humans remain inconclusive (1, 10–21).

As a dietary supplement, HMB is available as a calcium salt of HMB (HMB-Ca) or as a free acid form of HMB (HMB-FA) (8, 22, 23). There are only three studies comparing the kinetics of HMB-Ca and HMB-FA (22–24). Two studies by Fuller and colleagues (23, 24) indicate the superior bioavailability of HMB-FA over HMB-Ca, while the newest research by Ribeiro et al. (22) is in contrast to previous investigations. Nevertheless, based on the currently available literature, HMB-FA should be acknowledged as being more readily bioavailable compared to HMB-Ca. This may be of particular relevance in the context of acute, single-dose supplementation and the potential acute ergogenic effects of HMB.

It should be underlined that, until now, HMB supplementation has mostly been studied using relatively narrow dosage ranges, with the standard dose equal to 3 g_HMB_·day^−1^ often arbitrarily accepted as sufficient. Additionally, previous research has rarely applied individualized dosing strategies. A major limitation in this respect may also be HMB supply under usual training/lifestyle conditions, which may not have been sufficient to induce homeostasis disturbances necessary to stimulate adaptation. Moreover, most previous studies implemented 12-week supplementation periods. However, there are two previous studies (1, 15) that utilized short-term HMB supplementation and in which significant increases in FFM content were observed. In the study by Zając et al. (15), 30-day HMB supplementation resulted in decreases in fat mass (FM) and increases in FFM, while Nissen et al. (1) observed increases in FFM after 7 weeks of HMB supplementation in young trained males. Therefore, it was reasonable to examine whether higher and individualized doses would produce clearer effects on body mass and composition. It was also important to determine whether (a) a shorter supplementation period, combined with a higher dose, (b) the varied physiological status of participants (trained/untrained), and (c) the influence of usual training/lifestyle conditions or their combination with high-intensity functional training (HIFT)-induced stimulation would lead to significant effects. Combining a shorter supplementation period with higher doses of HMB may represent a particularly valuable strategy in the context of sports competition or nutritional recovery in the management of malnutrition. In both scenarios, rapid improvements in body composition, particularly increases in FFM, may translate into tangible and measurable benefits, such as enhanced physical performance or greater muscle strength. So far, there are no studies on HMB that addressed the described combination in the context of trained and untrained individuals. Moreover, short-term evaluation of HMB effectiveness is of practical importance for athletes. Athletes and coaches often aim for outcomes within relatively short training cycles of 6–8 weeks (e.g., training camps or preseason preparation). Thus, it is reasonable to verify whether HMB could be incorporated during specific (short-term) preparation phases.

The aim of this study was to evaluate the influence of HMB-FA supplementation – alone (for 3 weeks) and combined with HIFT (3 weeks) – in young to middle-aged men with different levels of training experience. We hypothesized that HMB-FA (90 mg_HMB_·kg_FFM_·day^−1^, split into two servings per day), considered the most bioavailable form of HMB at the time of the study design, would contribute to a reduction in body mass and improvements in body composition, specifically by decreasing fat mass (FM) and increasing FFM. These effects were expected to be enhanced by the addition of HIFT and be most visible in untrained individuals.

## 2 Materials and methods

### 2.1 Study design

**Figure 1:**
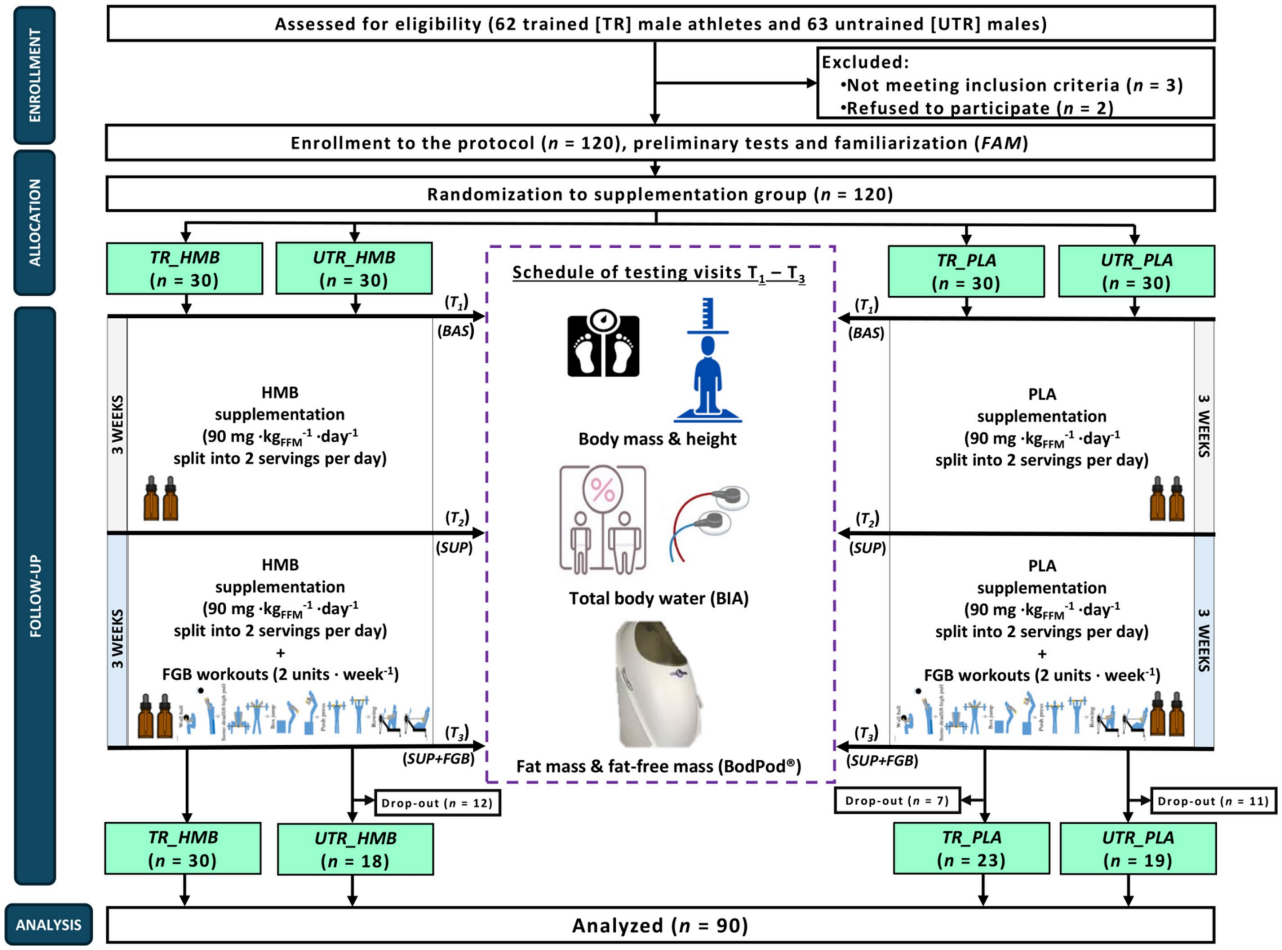
The flow chart of the study design. BAS, baseline; HMB, β-hydroxy-β-methylbutyrate; PLA, placebo; SUP, the first supplementation period during the usual training plan/lifestyle; SUP+FGB, the second period of the HMB/PLA treatment combined with the additional (in addition to their usual training plan/lifestyle) exercise stimuli in the form of two Fight Gone Bad (FGB) training units per week; T_1_-T_3_, study visits; TR, study participants categorized as ‘trained’; UTR, study participants categorized as ‘untrained’.

The study was a randomized, triple-blind, PLA-controlled, parallel-group trial and separated into two 3-week periods, of which the first one was the only supplementation period (*SUP*) – participants were ingesting either HMB or PLA in an individualized doses and were performing their usual training plan (trained) or lifestyle (untrained). The second period involved HMB/PLA treatment that was combined with the additional (in addition to their usual training plan/lifestyle) exercise stimuli in the form of two *Fight Gone Bad* (FGB) training units per week (*SUP+FGB*) (Figure 1). Apart from a familiarization visit (*FAM*), testing visits were performed at baseline (*BAS*) and after completion of the *SUP* and the *SUP + FGB* periods.

The main study was conducted in a few waves between September 2021 and June 2024 at the Department of Sports Dietetics (Poznan University of Physical Education, Poland) and the Sport Sciences–Biomedical Department (Charles University in Prague, Czech Republic). Due to the COVID-19 pandemic and sanitary and epidemiological measures, the period of study conduction was longer than firstly assumed (all research attempts made between March 2020 and August 2021 ended in failure due to lockdown or COVID-19 dropouts). The study protocol was reviewed and approved by the Bioethics Committee of the Regional Medical Chamber in Szczecin (No. 12/2022 from October 6, 2022), the Ethics Committee of the Faculty of Physical Education and Sport at Charles University (No. 243/2021 from November 5, 2021), and the Bioethics Committee at the Poznan University of Medical Sciences (No. 733/19 from June 19, 2019) and registered prospectively at ClinicalTrials.gov (NCT05444959; September 10, 2021). Written informed consent was obtained from all participants before their participation in the study began. All procedures were conducted in accordance with the ethical standards of the 1975 Declaration of Helsinki and its further updates. The study complies with the CONSORT statement for randomized trials, as shown in Supplementary material 1.

The G*Power software (version 3.1.9.4, Universität Düsseldorf, Germany) was used for *a priori* calculation of the sample size required to obtain a power of approximately 80% (*α* = 0.05) and large effect size $\eta_{p}^{2}=0.14.$ The required calculated total sample size suitable to detect a difference between three measurements and four groups for the analysis of variance with repeated measurements (RM ANOVA) within-between interaction was equal to 64 (16 per group). To account for possible dropouts (and an imbalanced dropout distribution across groups), 120 participants were initially enrolled in the study (30 per group).

### 2.2 Study participants

In total, 120 participants were enrolled in the study protocol, however there was a relatively high drop-out rate especially among untrained (UTR) individuals (Figure 1), Eventually, 90 participants completed the full protocol (Table 1), of which 53 were TR (29.1 ± 7.7 years; body mass [BM]: 84.8 ± 10.0 kg; FFM: 84.1 ± 5.1%) and 37 UTR participants (32.3 ± 7.6 years; BM: 90.1 ± 16.5 kg; FFM: 75.7 ± 7.7%). The eligibility criteria were male sex, aged 20–45 years, with an up-to-date medical clearance to practice sports. For TR individuals, they had to engage in regular physical activity (>250 min per week) and have training practice >10 years. UTR individuals had a lack of regular physical activity and no history of participation in competitive sports. The exclusion criteria were current injury, any health-related contraindication, declared general feeling of being unwell, being unwilling to follow the study protocol, a history of anabolic androgenic steroids or drugs use that may interfere with muscle mass control (e.g., corticosteroids) or affect physical performance, or the presence of infectious disease in the previous 4 weeks of the study. The TR participants enrolled into the protocol could be categorized to the Tier 2 and 3 (trained/developmental or highly trained/national level) category according to the latest training status classification framework by McKay et al. (25).

**Table 1:** Study participants.

Characteristics	Units	Trained	Untrained	*p*
*n*	–	53	37	–
Age	(years)	29.1 ± 7.7 (26.9–31.2)	32.3 ± 7.6 (29.8–34.8)	0.050
Height	(m)	1.82 ± 0.06 (1.80–1.84)	1.80 ± 0.08 (1.78–1.83)	0.306
Body mass	(kg)	84.8 ± 10.0 (82.0–87.5)	90.1 ± 16.5 (84.6–95.6)	0.061
BMI	(kg⋅m^2-1^)	25.6 ± 2.4 (24.9–26.2)	27.7 ± 4.7 (26.1–29.3)	**0.006**
FFM	(%)	84.1 ± 5.1 (82.7–85.5)	75.7 ± 7.7 (73.1–78.3)	**<0.001**
FFM	(kg)	71.2 ± 8.9 (68.8–73.7)	68.4 ± 10.3 (65.0–71.9)	0.176
FM	(%)	15.9 ± 5.1 (14.5–17.3)	24.3 ± 7.7 (21.7–26.9)	**<0.001**
FM	(kg)	13.6 ± 4.9 (12.2–14.9)	22.8 ± 10.5 (19.3–26.3)	**<0.001**
TBW	(%)	61.8 ± 3.8 (60.7–62.8)	56.7 ± 4.6 (55.2–58.2)	**<0.001**
TBW	(L)	52.3 ± 6.6 (50.4–54.1)	50.5 ± 5.9 (48.5–52.5)	0.200
FFM/FM	–	6.00 ± 2.45 (5.32–6.68)	3.69 ± 1.96 (3.03–4.34)	**<0.001**
TBW/FFM	–	0.73 ± 0.03 (0.73–0.74)	0.74 ± 0.06 (0.72–0.76)	0.369

The results are expressed as the mean ± standard deviation and 95% confidence interval (in parentheses). BMI, body mass index; FFM, fat-free mass; FM, fat mass; TBW, total body water.The bold values refer to statistically significant differences.

### 2.3 Study visits

In order to control for diurnal variability in the measured outcomes, all participant visits were initiated at the same time of day. In general, study appointments were scheduled during the morning hours. Two hours before each visit, participants consumed a standardized meal as previously described (containing 2 g_CHO_·kg_BM_^−1^, 30 g of protein and at least 7 mL_water_·kg_BM_^−1^) (26–28) and ingested the prescribed supplement 45 min before the start of test exercises (testing visit 2 and 3). Participants were asked to avoid caffeine intake 24 h prior to each visit at the laboratory. Furthermore, participants did not follow any specific nutritional strategies or make any changes to their usual diet (the habitual diet was monitored 48 h before *BAS*, *SUP*, and *SUP + FGB* visits *via* dietary recording using food diaries). The results of dietary recording are presented in Supplementary material 2.

During *BAS*, *SUP*, and *SUP+FGB*, participants BM, height, and body composition (FFM and FM) were assessed based on the air displacement plethysmography method using the BodPod^®^; (Cosmed, Italy) and their total body water (TBW) content was measured using electrical bioimpedance (BIA-101ASE; Akern, Italy); Figure 1). Exercise testing as well as blood and muscle analyses were conducted as part of the study; however, these aspects fall beyond the scope of the current article and will be presented separately.

### 2.4 Supplementation

#### 2.4.1 Blinding and randomization

The study participants were supplemented with either HMB or PLA for two 3-week periods. The study was triple-blind; thus, the study participants, researchers, and results assessors were not aware of the intervention (supplementation) received by each study participant. Randomization details were anonymized and revealed after protocol cessation. The randomization process was done by a staff member who did not directly participate in the investigations. FFM measured during *BAS* visit was utilized as a stratification factor between HMB and PLA within the TR and UTR groups.

#### 2.4.2 Dose calculation and preparation

HMB was applied in the form of free liquid *β*-hydroxy-β-methylbutyrate (Trec Nutrition Sp. z o.o., Poland). PLA was color-, taste-, and consistency-matched. Both preparations were provided in exactly the same brown 100 mL glass bottles with a dropper tip. Based on the density of both preparations and supplementation doses of HMB (90 mg·kg_FFM_^−1^·day^−1^), individual personalized total daily doses of HMB or PLA were calculated separately for each participant (based on FFM measured during the *BAS* visit) and expressed as number of drops per single portion. The calculations of individualized portions and labeling of the bottles with unique participant code numbers was carried out by a member of the research team who was not directly involved in conducting the study procedures. The individual total daily dose was split into two equal doses per day.

#### 2.4.3 Supplementation timing

Supplementation started on the day of the *BAS* visit, after completion of all procedures, under the supervision of researchers (participants were instructed on how to prepare the preparations for ingestion). On training days with one training session, participants were instructed to ingest the first portion of the supplement 45 min before the start of the training and the second one right after completion of the training. On days with two training sessions, each of two doses were taken 45 min before each training session. On testing days (*SUP* and *SUP+FGB* visits), participants ingested the supplemented preparation 45 min before the start of the exercise tests (incremental cycling tests to exhaustion) and the second right after completion of the visit. On non-training days, the first portion was taken right after waking up, and the second portion was taken before going to sleep. Each individual portion of the supplemented preparation was prepared by dissolving a precisely defined number of drops in 100–200 mL of plain water.

#### 2.4.4 Compliance monitoring

Compliance with HMB and PLA ingestion was monitored by researchers during and in-between testing visits, while participants were obligated to return the used bottles and submit a request if they were emptying the bottles between visits.

### 2.5 High-intensity exercise stimuli

During the second study period, in addition to supplementation, participants were obligated to introduce two FGB workouts to their usual training routine (TR group) or non-training routine (UTR group).

The FGB workout is a well-established benchmark in high-intensity functional training and was implemented in the present study as a standardized and multifunctional training session. The FGB protocol was chosen due to its specificity to the training modality and its well-documented structure and reliability, as confirmed in our previous research (29–31). Each participant completed the training session following a fixed and validated protocol. The total training duration was 17 min, consisting of three 5-min rounds, interspersed with two 1-min rest intervals. Each round included five exercises performed consecutively: Wall Ball Shots (9 kg [TR] or 6 kg [UTR] ball to a 3.0 m target); Sumo Deadlift High Pulls (barbell with weights: 35 kg [TR] or 15–20 kg [UTR]); Box Jumps (jumping on a box: 60 cm [TR] or 50 cm [UTR]); Push Presses (barbell with weights: 35 kg [TR] or 15–20 kg [UTR]), and rowing (damper setting 7, Ergometer Concept2 [Concept2, Inc., USA]). Participants performed each exercise for 1 min, aiming to complete as many valid repetitions (or calories, in the case of rowing) as possible. Transitions between exercises within the same round were immediate and without rest. All FGB workouts were performed under the supervision of experienced researchers at the Department of Sports Dietetics or the Sport Sciences–Biomedical Department. In participants assigned to the UTR group, exercise intensity and complexity were individually adapted to match each subject’s physical capacity. Modifications involved, for instance, adjusting box height, allowing step-ups instead of box jumps, and scaling resistance loads including barbells, plates, and medicine balls as described above.

To date there is no standardized workout / exercise stimuli validated in both trained and untrained individuals. The FGB workout was selected as a suitable exercise stimulus as none of the trained participants were regularly engaged in HIFT. The FGB provided a novel stimulus for trained individuals and an appropriate workload for untrained participants. HIFT training stresses both aerobic and anaerobic energy pathways and develops power, strength, flexibility, speed, endurance, agility, and coordination (32). HIFT is executed at a high intensity and emphasizes functional, multi-joint movements *via* endurance, strength, power, and speed-stimulating exercises (33, 34). Still, one of the most important strengths of HIFT is that the type and intensity of exercises can be adapted to the individual abilities of the participants. FGB is a validated test exercise (29). Our research team has great experience in utilizing FGB as test exercises in various supplementation and dietary interventions in athletes. In the case of untrained participants, standardization was based on encouraging them to perform at their maximal capacity (e.g., maximum lifted weight or maximum number of repetitions), which we consider to represent the strongest possible individual exercise stimulus at the time of the study execution.

### 2.6 Body mass and height

Body mass and height measurements were performed according to the recommendations as described previously (35) using a professional medical scale (WPT 60/150 OW, RADWAG^®^, Poland).

### 2.7 Fat-free mass and fat mass

FM and FFM were assessed by air displacement plethysmography (BodPod^®^, Cosmed, Italy) according to the recommendations described and applied previously (29, 36, 37). Body volume was assessed following a standardized protocol. The BodPod^®^ device was calibrated prior to each session using a 50-L cylinder in accordance with manufacturer guidelines. BM was measured using a calibrated digital scale immediately before the assessment. Participants wore minimal, tight-fitting clothing (swimsuit and swim cap) to minimize air displacement artifacts. Each single measurement was performed twice and, if the difference between measurements exceeded 150 mL, a third measurement was taken. FM percentage was calculated using the Siri equation (36). The reproducibility of BodPod^®^ measurements in our laboratory were previously verified and the results are published elsewhere (29, 38).

### 2.8 Total body water content

The TBW was assessed by bioelectric impedance with BIA-101ASE BIVA^®^ PRO (Akern, Italy) and BIATRODES™ electrodes (Akern, Italy) according to the standardized protocol and recommended measurement conditions (37, 39). The reproducibility of BIA measurements in our laboratory were previously verified and the results are published elsewhere (29, 38).

### 2.9 Statistical analysis

All variables were checked for a normal distribution with the Shapiro–Wilk test. The results are presented as the mean ± standard deviation (SD) and 95% confidence interval (CI). Baseline comparisons between TR and UTR were performed using t test for independent variables. Taking into account the robustness of the *F*-test in terms of Type 1 error, if the normality assumptions based on the Shapiro–Wilk test were violated, the kurtosis and skewness variables were also evaluated (40). A repeated measures ANOVA with Greenhouse–Geisser correction was conducted to examine the main and interaction effects of treatment (HMB/PLA) and training status (TR / UTR) factors over repeated measurements (*BAS, SUP*, and *SUP + FGB*). The analysis tested for within-subject effects, between-subject effects, and their interactions (*visit x treatment*; *visit x training status [TS]*; and *visit x treatment x TS*). Post-hoc comparisons were performed with a Bonferroni test. Effect size was expressed as $n_{p}^{2}$ (interpretation: <0.010 no effect, from 0.010 to 0.059 small effect, from 0.060 to 0.139 moderate effect and ≥0.140 large effect). Only participants who completed the full study protocol and with no missing data were considered in the statistical analysis. The data were analyzed using the STATISTICA 13.3 software (StatSoft Inc., Tulsa, OK, USA). Statistical significance was set at *p* < 0.05.

## 3 Results

**Figure 2:**
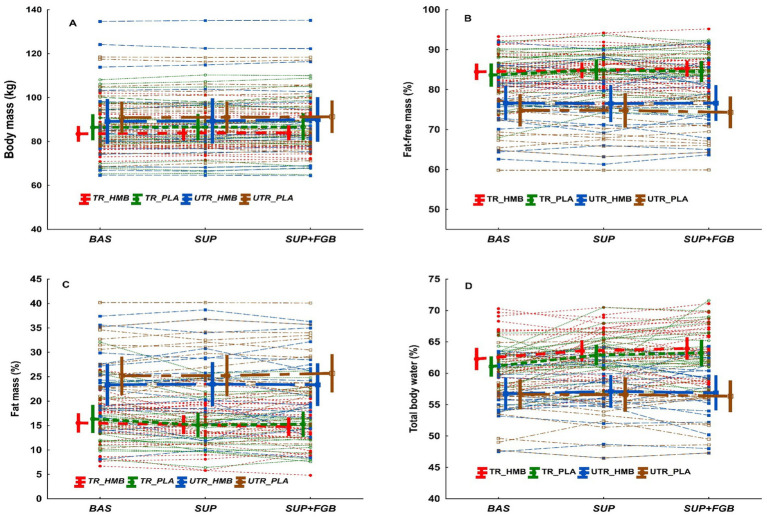
Spaghetti plot for individual changes in body mass **(A)**, fat-free mass **(B)**, fat mass **(C)**, and total body water **(D)**. The bold lines are means ± 95% CI.

There was a significant main effect of visit on BM and BMI (*p* = 0.013, $n_{p}^{2}$=0.049 and *p* = 0.023, $n_{p}^{2}$=0.044, respectively; Table 2), with BM and BMI being significantly higher at *SUP+FGB vs. BAS* (87.4 ± 13.3 *vs.* 87.0 ± 13.3 kg, *p* = 0.011 and 26.6 ± 3.6 *vs.* 26.5 ± 3.6 kg·m^-2-1^; *p* = 0.018). Individual changes in BM across study visits are presented in Figure 2A. However, there were no significant *visit x TS*, *visit x treatment*, or *visit x TS x treatment* interactions for BM or BMI.

**Table 2:** Body mass, body composition, and anthropometric indices.

Characteristics	Units	VISIT	*TRAINED*			*UNTRAINED*			*visit* *p* $n_{p}^{2}$ *1-β*	*visit x treatment* *p* $n_{p}^{2}$ *1-β*	*visit x TS* *p* $n_{p}^{2}$ *1-β*	*visit x treatment x TS* *p* $n_{p}^{2}$ *1-β*
			*HMB*	*PLA*	*p* *HMB vs. PLA* at *BAS*	*HMB*	*PLA*	*p* *HMB vs. PLA* at *BAS*				
*n*	**–**	**–**	30	23		18	19		–	–	–	–
Body mass	(kg)	*BAS*	83.5 ± 7.8 (80.6–86.4)	86.5 ± 12.4 (81.1–91.8)	0.286	89.2 ± 19.3 (79.6–98.8)	91.0 ± 13.8 (84.3–97.6)	0.747	**0.013** **0.049** **0.759**	0.406 0.010 0.204	0.550 0.007 0.148	0.884 0.001 0.068
		*SUP*	83.8 ± 7.2 (81.1–86.5)	86.6 ± 12.5 (81.2–92.0)	–	89.4 ± 19.4 (79.8–99.1)	91.0 ± 13.7 (84.4–97.6)	–				
		*SUP+FGB*	84.0 ± 7.4 (81.2–86.8)	86.7 ± 12.8 (81.1–92.2)	–	90.1 ± 19.1 (80.6–99.6)	91.3 ± 14.1 (84.5–98.1)	–				
BMI	(kg⋅m^-2-1^)	*BAS*	25.3 ± 1.8 (24.7–26.0)	25.9 ± 2.9 (24.7–27.2)	0.384	27.9 ± 4.4 (25.8–30.0)	27.5 ± 5.2 (24.9–30.1)	0.803	**0.023** **0.044** **0.705**	0.355 0.012 0.230	0.568 0.007 0.143	0.851 0.002 0.075
		*SUP*	25.5 ± 1.7 (24.8–26.1)	26.0 ± 2.9 (24.7–27.2)	–	27.9 ± 4.3 (25.8–30.0)	27.5 ± 5.1 (25.0–30.1)	–				
		*SUP + FGB*	25.5 ± 1.7 (24.9–26.1)	26.0 ± 3.0 (24.7–27.3)	–	28.0 ± 4.3 (25.9–30.0)	27.8 ± 5.0 (25.3–30.3)	–				
FFM	(%)	*BAS*	84.5 ± 4.5 (82.8–86.1)	83.6 ± 5.9 (81.1–86.2)	0.575	76.6 ± 8.0 (72.7–80.6)	74.8 ± 7.6 (71.2–78.5)	0.482	0.312 0.013 0.255	0.358 0.012 0.228	0.075 0.030 0.519	0.702 0.004 0.106
		*SUP*	84.8 ± 4.4 (83.2–86.5)	84.9 ± 5.1 (82.7–87.1)	–	76.5 ± 8.5 (72.3–80.8)	74.8 ± 8.2 (70.8–78.7)	–				
		*SUP + FGB*	85.2 ± 4.4 (83.6–86.9)	84.5 ± 5.3 (82.2–86.8)	–	76.6 ± 8.2 (72.5–80.7)	74.3 ± 7.5 (70.7–77.9)	–				
FFM	(kg)	*BAS*	70.5 ± 7.4 (67.7–73.2)	72.2 ± 10.7 (67.6–76.8)	0.494	67.2 ± 9.8 (62.4–72.1)	69.6 ± 11.0 (64.3–74.9)	0.501	**0.018** **0.046** **0.722**	0.294 0.014 0.266	0.103 0.026 0.463	0.623 0.005 0.127
		*SUP*	71.0 ± 6.7 (68.6–73.5)	73.3 ± 10.0 (68.9–77.6)	–	67.3 ± 10.1 (62.3–72.3)	69.6 ± 11.0 (64.2–74.9)	–				
		*SUP + FGB*	71.5 ± 6.7 (69.1–74.0)	73.2 ± 10.7 (68.6–77.9)	–	67.9 ± 9.5 (63.2–72.6)	69.3 ± 11.3 (63.9–74.8)	–				
FM	(%)	*BAS*	15.5 ± 4.5 (13.9–17.2)	16.4 ± 5.9 (13.8–18.9)	0.575	23.4 ± 8.0 (19.4–27.3)	25.2 ± 7.6 (21.5–28.8)	0.482	0.274 0.015 0.280	0.434 0.010 0.192	0.050 0.034 0.583	0.644 0.005 0.121
		*SUP*	15.2 ± 4.4 (13.5–16.8)	15.1 ± 5.1 (12.9–17.3)	–	23.5 ± 8.5 (19.2–27.7)	25.2 ± 8.2 (21.3–29.2)	–				
		*SUP + FGB*	14.8 ± 4.4 (13.1–16.4)	15.3 ± 5.4 (13.0–17.6)	–	23.4 ± 8.2 (19.3–27.5)	25.7 ± 7.5 (22.1–29.3)	–				
FM	(kg)	*BAS*	13.0 ± 4.0 (11.5–14.5)	14.3 ± 5.9 (11.7–16.8)	0.351	21.9 ± 11.6 (16.2–27.7)	23.6 ± 9.6 (18.9–28.2)	0.644	0.542 0.007 0.151	0.494 0.008 0.168	**0.037** **0.038** **0.629**	0.645 0.005 0.121
		*SUP*	12.8 ± 4.0 (11.3–14.3)	13.3 ± 5.3 (11.0–15.6)	–	22.1 ± 11.8 (16.2–28.0)	23.6 ± 10.3 (18.7–28.6)	–				
		*SUP + FGB*	12.5 ± 4.0 (11.0–14.0)	13.4 ± 5.5 (11.0–15.8)	–	22.1 ± 11.7 (16.3–28.0)	24.2 ± 10.0 (19.4–29.0)	–				
TBW	(%)	*BAS*	62.3 ± 4.1 (60.7–63.8)	61.1 ± 3.2 (59.7–62.5)	0.254	56.7 ± 4.8 (54.4–59.1)	56.6 ± 4.5 (54.5–58.8)	0.944	**0.002** **0.072** **0.909**	0.936 0.000 0.051	**0.002** **0.070** **0.903**	0.653 0.005 0.121
		*SUP*	63.6 ± 3.9 (62.1–65.0)	62.9 ± 3.3 (61.5–64.3)	–	57.1 ± 4.8 (54.7–59.5)	56.6 ± 5.2 (54.1–59.1)	–				
		*SUP + FGB*	64.0 ± 4.2 (62.4–65.5)	63.3 ± 4.3 (61.4–65.1)	–	56.9 ± 5.2 (54.3–59.4)	56.3 ± 4.8 (54.1–58.6)	–				
TBW	(L)	*BAS*	51.9 ± 5.7 (49.8–54.0)	52.7 ± 7.8 (49.4–56.1)	0.660	49.9 ± 7.1 (46.4–53.4)	51.1 ± 4.7 (48.8–53.4)	0.545	**0.000** **0.110** **0.989**	0.941 0.001 0.059	**0.002** **0.070** **0.902**	0.661 0.005 0.116
		*SUP*	53.3 ± 5.1 (51.4–55.2)	54.5 ± 8.0 (51.0–57.9)	–	50.2 ± 7.1 (46.7–53.8)	51.1 ± 4.9 (48.7–53.4)	–				
		*SUP + FGB*	53.7 ± 5.2 (51.7–55.6)	54.7 ± 8.4 (51.1–58.4)	–	50.4 ± 6.7 (47.1–53.8)	51.1 ± 5.3 (48.5–53.6)	–				
FFM/FM	–	*BAS*	6.12 ± 2.61 (5.14–7.09)	5.85 ± 2.27 (4.86–6.83)	0.696	3.93 ± 2.29 (2.79–5.07)	3.46 ± 1.63 (2.67–4.24)	0.471	0.108 0.026 0.456	0.714 0.004 0.103	0.091 0.028 0.485	0.880 0.001 0.069
		*SUP*	6.41 ± 3.22 (5.21–7.61)	6.41 ± 2.70 (5.24–7.58)	–	3.95 ± 2.17 (2.87–5.03)	3.52 ± 1.69 (2.71–4.34)	–				
		*SUP + FGB*	6.59 ± 3.24 (5.38–7.80)	6.39 ± 2.74 (5.21–7.58)	–	3.96 ± 2.28 (2.83–5.10)	3.34 ± 1.50 (2.62–4.06)	–				
TBW/FFM	–	*BAS*	0.74 ± 0.04 (0.72–0.75)	0.73 ± 0.03 (0.72–0.74)	0.526	0.74 ± 0.03 (0.73–0.76)	0.74 ± 0.08 (0.71–0.78)	0.970	**0.032** **0.039** **0.651**	0.569 0.007 0.143	0.111 0.025 0.450	0.922 0.001 0.062
		*SUP*	0.75 ± 0.03 (0.74–0.76)	0.74 ± 0.03 (0.73–0.76)	–	0.75 ± 0.05 (0.73–0.77)	0.74 ± 0.07 (0.71–0.77)	–				
		*SUP+FGB*	0.75 ± 0.03 (0.74–0.76)	0.75 ± 0.03 (0.73–0.76)	–	0.74 ± 0.03 (0.73–0.76)	0.75 ± 0.08 (0.71–0.78)	–				

The results are expressed as the mean ± standard deviation and 95% confidence interval (in parentheses). BAS, baseline; BMI, body mass index; FFM, fat-free mass; FM, fat mass; HMB, β-hydroxy-β-methylbutyrate; PLA, placebo; SUP, the first period of the supplementation period during the usual training plan/lifestyle; SUP+FGB, the second period of the HMB/PLA treatment combined with the additional (in addition to their usual training plan/lifestyle) exercise stimuli in the form of two Fight Gone Bad (FGB) training units per week; TBW, total body water; TS, training status. Bold values refer to statistically significant changes/interactions.

Percentage FFM was unchanged during the full study protocol regardless of the implemented treatment or training status (Table 2). Individual changes in FFM (%) across study visits are presented in Figure 2B. There was a significant main effect of visit on FFM in kg (*p* = 0.018, $n_{p}^{2}$=0.046; Table 2), with FFM being significantly higher at *SUP+FGB* vs. *BAS* (70.8 ± 9.5 *vs.* 70.1 ± 9.6 kg; *p* = 0.005). When analyzing each subgroup separately, an increase in FFM was observed in *TR_HMB*, *TR_PLA*, and *UTR_HMB* but not in *UTR_PLA* (Table 2). However, there were no significant *visit x treatment*, *visit x TS*, or *visit x treatment x TS* interactions for FFM.

FM (%) was unchanged during the full study protocol regardless of the implemented treatment or TS (Table 2). Individual changes in FM (%) across study visits are presented in Figure 2C. There was no effect of visit on FM in kg, however there was a significant effect with *visit x TS* interaction (*p* = 0.037, $n_{p}^{2}$=0.038; Table 2), with FM decreasing consecutively in *TR_HMB* and *TR_PLA* but increasing in *UTR_PLA*. However, there were no significant *visit x treatment* or *visit x treatment x TS* for FM in kg.

There were significant main effects of visit on TBW percentage (*p* = 0.002, $n_{p}^{2}$=0.072) and liters (L) (*p* < 0.001, $n_{p}^{2}$=0.110; Table 2). Percentage TBW was significantly higher at *SUP* (*p* = 0.002) and *SUP+FGB* (*p* < 0.001) *vs. BAS* (60.6 ± 5.3 and 60.8 ± 5.7 *vs.* 59.7 ± 4.8%, respectively) and, similarly, TBW in L was significantly higher at *SUP* (*p* < 0.001) and *SUP+FGB* (*p* < 0.001) *vs. BAS* (52.5 ± 6.4 and 52.7 ± 6.6 *vs.* 51.5 ± 6.4 L, respectively). When analyzing each subgroup separately, an increase in TBW was observed in *TR_HMB*, *TR_PLA*, and *UTR_HMB* but not in *UTR_PLA* (Table 2). Individual changes in TBW (%) across study visits are presented in Figure 2D. Moreover, there were significant *visit x TS* interactions for TBW in percentage (*p* = 0.002, $n_{p}^{2}$=0.070) and L (*p* = 0.002, $n_{p}^{2}$=0.070; Table 2). The interactions clearly indicated greater increases in TBW in consecutive study visits in TR *vs.* UTR individuals (regardless implemented treatment). However, there were no significant *visit x treatment* or *visit x TS x treatment* interactions for TBW (% and L).

Furthermore, FFM/FM was unchanged during the study protocol regardless of the implemented treatment or TS (Table 2). However, there was a significant main effect of *visit* on TBW/FFM (*p* = 0.032, $n_{p}^{2}$=0.039; Table 2), with TBW/FFM being higher at *SUP* and *SUP+FGB* vs. *BAS* (*SUP*: 0.75 ± 0.04 *vs.* 0.74 ± 0.05, *p* = 0.037; *SUP+FGB*: 0.75 ± 0.05 *vs.* 0.74 ± 0.05, *p* = 0.018, respectively).

## 4 Discussion

To the best of our knowledge, this is the first study to implement an individually adjusted fat-free mass dose of free liquid HMB as high as 90 mg·kg_FFM_^−1^·day^−1^ in trained and untrained male participants within a single study protocol. The effectiveness of HMB was evaluated in terms of their usual training schedule (or no training and customary lifestyle in UTR) and in conjunction with additional HIFT stimuli (TR and UTR). The primary results of the current study revealed that two 3-week periods of HMB supplementation (in total 6 weeks) had no significant effect on changes in body mass, fat-free mass, fat mass, or total body water content. Still, the main effect of visit (*BAS*, *SUP*, and *SUP+FGB*) was observed for BM and BMI, FFM (kg), TBW (% and L), and TBW/FFM. Moreover, there were significant *visit x training status* interactions for FM (kg) and TBW (% and L).

Taking into account the FFM of study participants, the actual implemented HMB doses ranged from 4.8 to 7.8 g·day^−1^. The most widely used and best studied dose of HMB in the context of body mass and composition seems to be 3 g_HMB_·day^−1^ (8). Still, earlier studies on the impact of HMB supplementation on body mass and composition provided inconclusive results. The results of our previous studies contrast with the current findings. In those crossover studies, we noted that 12 weeks of supplementation with 3 g_HMB-Ca_·day^−1^ led to a significant increase in FFM and a reduction in FM in trained combat sport athletes aged 22.8 ± 6.1 years (18); in elite male rowers aged 17–22 years, the same supplementation protocol resulted in a significant decrease in FM but no changes in FFM (19). Nevertheless, our current and previous supplementation protocols differ substantially in terms of duration, dosage, and the form of the supplement. Despite the use of a higher dose and a more bioavailable form of the supplement, the duration of supplementation was markedly shorter in the current study, which might partly explain the lack of recognizable changes. In the study by Kreider et al. (10), 28-day supplementation with 3 or 6 g_HMB-Ca_·day^−1^ in young resistance-trained individuals had no effect on lean body mass (LBM) or FM. Similarly, Ransone et al. (14) observed that four-week supplementation with 3 g_HMB-Ca_·day^−1^ had no effect on skin folds in young college football players; in the study by Slater et al. (13), the same dose was unable to evoke alternations in body composition after 6 weeks in highly trained water polo players and rowers. Teixeira et al. (41) found no effect of either 3 g_HMB-Ca_·day^−1^ or 3 g_HMB-FA_·day^−1^ after eight weeks of supplementation on body composition in trained males (31.7 ± 7.6 years). Surprisingly, in contrast to the aforementioned studies, Zając et al. (15) reported an impressive increase in FFM (~2 kg) and a decrease in FM (~1.3%) in young trained basketball players after only 30 days of supplementation. However, the dose and form of the supplement were not clearly specified in that study. The pioneering investigations by Nissen et al. (1) revealed a significant increase in FFM (1.9 kg) and no effect on FM after 7 weeks of supplementation with 3 g_HMB-Ca_·day^−1^ in young trained men. In addition, Thomson et al. (17) revealed that a similar HMB dose led to a significant decrease in skinfold sum after 9 weeks of supplementation. Nevertheless, Fernández-Landa et al. (42) observed no changes in skinfold thickness after 10 weeks of supplementation with the discussed dose. The remaining studies available in this area employed a 12-week supplementation protocol with a daily dose of 3 g_HMB-Ca_·day^−1^ (20, 43) or 3 g_HMB-FA_·day^−1^ (43, 44). While Wilson et al. (44) observed a significant increase in LBM (~5.3 kg) and decrease in FM (~3.7 kg), Tritto et al. (43) and McIntosh et al. (20) found no effect of supplementation on body composition outcomes.

Apart from the relatively short supplementation period, another reason for the null findings in the current study may be the lack of an additional stressor, namely an energy deficit. In the study, both trained and untrained participants were required to maintain their habitual food intake throughout the protocol. Based on dietary records collected 48 h before each study visit, we did not observe changes in energy intake (kcal⋅day^−1^) in any of the studied subgroups. Introducing another confounding factor, such as an energy deficit, in the current protocol could have diminished the potential effect of the implemented HIFT stimuli. Considering the previously mentioned effect of HMB intake on MPS and MPB, a more pronounced effect on FFM changes (even with the same supplementation duration) would likely be observed under a hyperenergetic diet. From a practical sports perspective, it may be reasonable to apply HMB supplementation during weight reduction phases (e.g., in combat sports due to weight categories) to prevent body mass losses resulting from FFM decreases. Similarly, HMB supplementation during weight loss in recreationally active individuals may be worth considering to promote so-called high-quality weight loss—a reduction in FM while preserving and/or increasing FFM content.

It should be noted that there was one previous study by Gallagher et al. (11) that implemented individualized doses equal to 38 or 76 mg_HMB-Ca_·kg_BM_·day^−1^ (or placebo). In that study, untrained college-age males were supplemented with HMB for 8 weeks in addition to resistance training (3 workouts·week^−1^; in total 28 workouts during the full study protocol). It was surprisingly found that the dose of 38 mg_HMB_·kg_BM_·day^−1^ was effective in evoking increases in FFM (+1.9 kg of FFM). Compared to the current study, which found no effect of HMB supplementation on FFM in untrained young to middle-aged males, the duration of supplementation in the study by Gallagher et al. was only 2 weeks longer; however, the overall training volume was considerably higher. Regarding other studies conducted on healthy, untrained men, Nissen et al. (1) and Jówko et al. (12) showed no statistically significant changes in body composition following 3 weeks of HMB supplementation at doses between 1.5 and 3 g_HMB-Ca_·kg_BM_·day^−1^. However, longer duration studies (12 weeks, 3 g_HMB-Ca_·kg_BM_·day^−1^) revealed more promising results (16, 21). Stahn et al. (21) found medium-to large effect sizes for the effect of HMB on total and segmental FFM increase; Kraemer et al. (16) found an increase in LBM (~ + 5 kg) and reduction in FM (~ − 2%).

Based on the results of the current investigation and our previous and other studies, it must be stated that, regardless of training status, the duration of supplementation seems to be crucial for evoking favorable changes in body composition after HMB supplementation. Thus, it must be clearly emphasized that an increased dosage of HMB cannot substitute for the requirement of a sufficiently prolonged supplementation period to elicit a biological response. Consequently, when aiming to enhance body composition through HMB supplementation in professional sport, its implementation should be strategically integrated into the training macrocycle to ensure maximal effectiveness during the athlete’s key preparation or competition phases.

In the current study, we observed an interesting *visit x training status* interaction for FM in kg. During the study protocol, the following changes in FM content were observed in particular groups: *TR_HMB*: ~ − 0.5 kg, *TR_PLA*: ~ − 0.9 kg, *UTR_HMB*: ~ + 0.2 kg, and *UTR_PLA*: ~ + 0.6 kg. The latter may indicate that supplementation with HMB in UTR exerted a protective effect against further increases in FM, which would likely have occurred in the absence of intervention. Simultaneously, in *UTR_PLA*, in contrary to the remaining subgroups, a slight decrease in FFM content within the study protocol was observed (~ − 0.3 kg). Interestingly, regardless of the implemented treatment, TR individuals in contrary to UTR individuals tended to improve their hydration status based on TBW content (*visit x training status* interaction). Changes in hydration status may occur as a secondary effect of increased FFM, particularly skeletal muscle tissue, which constitutes the primary reservoir of water in the human organism. Notably, the *UTR_PLA* group was the only subgroup in which a slight decrease in TBW percentage was observed. Based on the described observations, from the point of view of slowing undesirable changes in body composition (i.e., an increase in FM and a decrease in FFM), it may be assumed that UTR individuals could benefit more from HMB supplementation than TR individuals. The differences in the response to HMB supplementation between TR and UTR individuals would probably be more pronounced during a deliberate body mass reduction process. Still, the direction of the presumed differences remains to be disclosed, since UTR individuals may introduce an energy deficit and increase energy expenditure during weight loss, whereas athletes (who already experience high exercise-related energy expenditure) should rely more on changes in dietary behavior and food intake when considering safe and allowed methods of body mass reduction.

A key strength of the present study lies in the individualized supplementation protocol. Although only a limited number of previous studies have adopted such an approach, the International Society of Sports Nutrition’s position on HMB supplementation (8) explicitly indicate HMB dosing based on body mass—38 mg_HMB_⋅kg_BM_^−1^⋅day^−1^ (and in combination with exercise training)—to potentially improve body composition. This methodological consideration enhances the physiological relevance and precision of the intervention. The approach implemented in our study is in line with the newest recommendations. Another notable strength is the inclusion of both TR and UTR individuals within the same experimental protocol, allowing for broader applicability of the findings across populations with different training statuses. Furthermore, the use the FGB (a standardized HIFT unit (29)) as the additional exercise/lifestyle stimulus provided a consistent and replicable model of physical effort. What is more, all of the workouts performed by the study participants within the study protocol during the SUP + FGB period were strictly supervised by the experienced research team members. The application of the liquid free acid form of HMB allowed for precise individualization of the administered doses across participants. Another strength of this study is the use of air displacement plethysmography (BodPod^®^), a validated and reliable method for assessing FM and FFM, which ensured high accuracy in body composition measurements and reduced potential methodological bias.

Nonetheless, the study has limitations that warrant consideration. The relatively short duration of supplementation (6 weeks in total) and the limited number of training sessions during the *SUP+FGB* period (six FGB training units) may have attenuated the potential effects of HMB on body composition. At the same time, this limitation reveals an important insight: a high, acute (‘loading’)/medium-term chronic dose of HMB, even when individualized, is insufficient to induce measurable changes in FFM or FM over a short time frame. These findings emphasize the need to incorporate HMB supplementation into longer-term training programs or more severe/high-intensity training units concentrated in short time intervals to realize its full biological potential.

Additionally, we are aware that evaluating dietary intake for more than 48 h before each study visit might have made dietary control even more adequate. However, it needs to be emphasized that study participants were repeatedly instructed to keep their usual food intake throughout the entire study protocol. The intention was to ensure participants did not focus too much on consumed foods and drinks and as a result markedly change their food choice and, eventually, energy and macronutrients intake. Such changes would introduce additional confounding factors into the protocol (apart from supplementation and HIFT stimuli). We did not observe significant changes in energy value between study visits in any of the studied subgroups. There were no changes in protein, carbohydrate, or fat intake that might have affected the effectiveness of HMB supplementation and HIFT stimuli. Protein intake among TR study participants fluctuated around 2 g·kg^−1^·day^−1^, and among UTR study participants around 1.24–1.31 g·kg^−1^·day^−1^. Thus, dietary recommendations for protein intake were met in both TR and UTR study participants. According to the meta-analysis by Holland et al. (45), HMB may have a small, positive impact on FFM in athletes, although this seems specific to when protein intake is suboptimal (<1.6 g·kg^−1^·d^−1^). Because of this, a more pronounced effect of HMB supplementation would be more probable in UTR study participants.

The possibility of the placebo effect cannot be fully excluded. However, self-unblinding of the received treatment was not a reason for a possible placebo effect: the HMB and PLA preparations were identical in taste, texture, look, and packaging. Further, the FGB workouts were always supervised by the same qualified research team members—each participant was always equally motivated and encouraged by the supervisors to perform the workout as good as possible according to their individual capabilities. The main reason for the placebo effect may be related to the behaviors presented by the study participants outside the laboratory, e.g., by paying more attention to the type and amount of consumed food or intentionally or unintentionally undertaking more physical activity. Still, no changes in energy values or macronutrients from their habitual diet were observed throughout the study protocol. Thus, if the placebo effect occurred, it affected participants from HMB / PLA treatments in the same manner and eventually did not interfere with the final conclusions of the effect of HMB ingestion and HIFT stimuli on body mass and body composition. The placebo effect might have improved the effects evoked by the implemented interventions, although the magnitude of the effect is supposed to be equal in both treatment groups.

Although the study experienced a relatively high dropout rate, particularly among UTR participants (23 participants dropped out), this is not unexpected, as such individuals may perceive lower personal benefit from participating in experimental protocols (specially in combination with strenuous exercises) that focus on body composition or physical capacity compared to TR individuals (seven participants dropped out) who often associate such changes with improved performance outcomes. Nevertheless, despite the dropout, the final sample size, including all experimental subgroups, exceeded the minimum sample size (*n* = 16 per group) calculated *a priori* in the power analysis, ensuring sufficient statistical power for the primary comparisons.

It needs to be mentioned that the results of the current study refer solely to young and middle-aged trained and untrained males and cannot be extrapolated to other population groups, e.g., women or older adults. Thus, future studies must include other population groups that could benefit from HMB supplementation. Moreover, future studies must incorporate additional biomarkers, e.g., muscle damage, inflammation, or hormonal response, to comprehensively examine the effects of HMB from multiple angles.

To summarize, in the current randomized parallel-group placebo-controlled clinical trial, we evaluated the effects of an individualized, high-dose HMB supplementation protocol (90 mg·kg_FFM_^−1^·day^−1^) in both trained and untrained males within a unified experimental design (3 weeks of exclusive supplementation followed by 3 weeks of supplementation with HIFT stimuli). Despite the use of a more bioavailable form of HMB (free acid), a precisely individualized high dosing strategy, and HIFT stimuli, no significant changes in body mass, fat mass, fat-free mass, or total body water content were observed after two 3-week supplementation periods. These findings suggest that, even when high HMB doses are applied, a relatively short supplementation period is insufficient to induce meaningful changes in body composition. Importantly, while statistical significance was not achieved for body composition changes, the observed patterns and effect directions may still carry practical implications for training and supplementation strategies, particularly when integrated over longer timeframes. When placed in the context of prior research, our findings reinforce the notion that duration of supplementation is a critical determinant of HMB efficacy. Earlier studies demonstrating positive effects typically involved protocols lasting 8–12 weeks, unless the dose was about 3 g_HMB-Ca / HMB-FA_·day^−1^. Consequently, it must be clearly emphasized that increasing the HMB dose cannot compensate for the requirement of sufficient supplementation time.

## Data Availability

The original contributions presented in the study are included in the article/Supplementary material, further inquiries can be directed to the corresponding author.

## Supplementary material

SUPPLEMENTARY MATERIAL 1 CONSORT checklist.

SUPPLEMENTARY MATERIAL 2 Nutritional value of their habitual diet.
