# Supplementary material for: High-dose medium-term HMB supplementation did not trigger body composition changes in trained and untrained males under usual conditions or high-intensity functional exercise
Source: Front Nutr. 2025 Sep 24;12:1681465. doi: 10.3389/fnut.2025.1681465 (PMC12505008; doi:10.3389/fnut.2025.1681465)
Supplement: SUPPLEMENTARY MATERIAL 2 — Nutritional value of their habitual diet. [file Table_2.docx]

Supplementary Material 2

High-Dose Medium-Term HMB Supplementation did not Trigger Body Composition Changes in Trained and Untrained Males Under Usual Conditions or High-Intensity Functional Exercise

**Krzysztof Durkalec-Michalski ^1-3*^, Magdalena Czlapka-Matyasik ^2,4^, Tomasz Podgórski ^3,5^, Małgorzata Marchelek-Myśliwiec ^6^, Paulina M. Nowaczyk ^1-3^**

^1^ Department of Sports Dietetics, Poznan University of Physical Education, Poznań, Poland

^2^ Polish Society of Nutritional Sciences, Poland

^3^ Sport Sciences–Biomedical Department, Faculty of Physical Education and Sport, Charles University, Charles University, Prague, Czech Republic

^4^ Department of Human Nutrition and Dietetics, Poznań University of Life Sciences, Poznań, Poland

^5^ Department of Biochemistry, Poznan University of Physical Education, Poznań, Poland

^6^ Department of Nephrology, Transplantology and Internal Medicine, Pomeranian Medical University, Szczecin, Poland

*** Correspondence:**Krzysztof Durkalec-Michalski
durkalec-michalski@awf.poznan.pl

**Supplementary material 2.** Nutritional value of habitual diet

|  |  |  | ***TRAINED*** | | ***UNTRAINED*** | |
| --- | --- | --- | --- | --- | --- | --- |
| **Characteristics** | **Units** | **VISIT** | ***HMB*** | ***PLA*** | ***HMB*** | ***PLA*** |
| ***n*** | **-** | **-** | 30 | 23 | 18 | 19 |
| **Energy** | **(kcal∙day^-1^)** | ***BAS***  ***SUP***  ***SUP+FGB***  ***p;*** $\boldsymbol{n}_{\boldsymbol{p}}^{\boldsymbol{2}}$ | 3329 ± 329  (3206 – 3452)  3425 ± 323  (3304 – 3545)  3347 ± 321  (3227 – 3467)  0.770; 0.085 | 3393 ± 412  (3215 – 3571)  3496 ± 465  (3294 – 3697)  3405 ± 422  (3222 – 3587)  0.143; 0.085 | 2644 ± 340  (2475 – 2814)  2796 ± 319  (2637 – 2954)  2685 ± 328  (2522 – 2848)  0.052; 0.159 | 2775 ± 368  (2598 – 2952)  2823 ± 300  (2679 – 2968)  2794 ± 363  (2619 – 2968)  0.709; 0.019 |
| **Protein** | **(%)** | ***BAS***  ***SUP***  ***SUP+FGB***  ***p;*** $\boldsymbol{n}_{\boldsymbol{p}}^{\boldsymbol{2}}$ | 19.6 ± 3.1  (18.4 – 20.7)  19.7 ± 2.9  (18.6 – 20.7)  19.6 ± 3.0  (18.5 – 20.7)  0.571; 0.019 | 19.9 ± 2.5  (18,8 – 20.9)  20.1 ± 2.5  (19.0 – 21.2)  20.0 ± 2.5  (18.9 – 21.0)  0.142; 0.085 | 16.4 ± 2.1  (15.4 – 17.4)  16.3 ± 1.7  (15.5 – 17.2)  16.5 ± 2.0  (15.6 – 17.5)  0.590; 0.031 | 16.3 ± 2.5  (15.1 – 17.5)  16.6 ± 2.4  (15.4 – 17.8)  16.4 ± 2.4  (15.3 – 17.6)  0.118; 0.112 |
| **Protein** | **(g∙ kg^-1^∙day^-1^)** | ***BAS***  ***SUP***  ***SUP+FGB***  ***p;*** $\boldsymbol{n}_{\boldsymbol{p}}^{\boldsymbol{2}}$ | 1.97 ± 0.41  (1.81 – 2.12)  2.02 ± 0.37  (1.88 – 2.16)  1.97 ± 0.41  (1.82 – 2.12)  0.157; 0.062 | 1.97 ± 0.38  (1.81 – 2.14)  2.04 ± 0.32  (1.91 – 2.18)  1.98 ± 0.36  (1.83 – 2.14)  0.088; 0.105 | 1.24 ± 0.24  (1.12 – 1.36)  1.31 ± 0.27  (1.18 – 1.45)  1.26 ± 0.24  (1.14 – 1.38)  0.045; 0.166 | 1.26 ± 0.26  (1.13 – 1.38)  1.30 ± 0.24  (1.19 – 1.42)  1.27 ± 0.26  (1.15 – 1.40)  0.379; 0.053 |
| **Carbohydrate** | **(%)** | ***BAS***  ***SUP***  ***SUP+FGB***  ***p;*** $\boldsymbol{n}_{\boldsymbol{p}}^{\boldsymbol{2}}$ | 55.1 ± 3.5  (53.7 – 56.4)  55.1 ± 2.9  (54.0 – 56.2)  55.0 ± 3.3  (53.8 – 56.2)  0.834; 0.006 | 55.3 ± 3.1  (53.9 – 56.7)  55.2 ± 2.9  (54.0 – 56.4)  55.3 ± 3.0  (54.0 – 56.5)  0.753; 0.013 | 51.9 ± 5.8  (49.0 – 54.7)  52.4 ± 5.9  (49.5 – 55.3)  52.5 ± 6.1  (49.5 – 55.5)  0.430; 0.048 | 52.1 ± 5.6  (49.4 – 54.8)  52.3 ± 5.3  (49.8 – 54.9)  52.2 ± 5.4  (49.6 – 54.8)  0.775; 0.014 |
| **Carbohydrate** | **(g∙ kg^-1^∙day^-1^)** | ***BAS***  ***SUP***  ***SUP+FGB*** | 5.67 ± 0.60  (5.44 – 5.89)  5.64 ± 0.54  (5.44 – 5.84)  5.50 ± 0.62  (5.27 – 5.73)  0.066; 0.090 | 5.63 ± 0.65  (5.35 – 5.91)  5.62 ± 0.68  (5.32 – 5.91)  5.47 ± 0.62  (5.20 – 5.74)  0.193; 0.072 | 4.15 ± 0.68  (3.82 – 44.9)  4.19 ± 0.70  (3.84 – 4.54)  4.00 ± 0.68  (3.66 – 4.34)  0.136; 0.111 | 4.09 ± 0.62  (3.79 – 4.39)  4.11 ± 0.63  (3.80 – 4.41)  4.03 ± 0.60  (3.74 – 4.31)  0.692; 0.020 |
| **Fat** | **(%)** | ***BAS***  ***SUP***  ***SUP+FGB***  ***p;*** $\boldsymbol{n}_{\boldsymbol{p}}^{\boldsymbol{2}}$ | 24.2 ± 4.6  (22.5 – 25.9)  24.6 ± 4.1  (23.1 – 26.1)  24.4 ± 4.3  (22.8 – 26.0)  0.133; 0.067 | 23.3 ± 4.0  (21.6 – 25.0)  23.4 ± 3.5  (21.9 – 24.9)  23.3 ± 3.5  (21.8 – 24.9)  0.980; 0.001 | 30.6 ± 6.0  (27.6 – 33.5)  30.4 ± 6.4  (27.2 – 33.6)  29.9 ± 6.3  (26.8 – 33.1)  0.485; 0.042 | 30.3 ± 5.4  (27.7 – 32.9)  29.8 ± 5.4  (27.2 – 32.4)  30.3 ± 5.1  (27.8 – 32.8)  0.447; 0.044 |
| **Fat** | **(g∙ kg^-1^∙day^-1^)** | ***BAS***  ***SUP***  ***SUP+FGB***  ***p;*** $\boldsymbol{n}_{\boldsymbol{p}}^{\boldsymbol{2}}$ | 1.07 ± 0.21  (0.99 – 1.15)  1.12 ± 0.19  (1.04 – 1.19)  1.08 ± 0.20  (1.00 – 1.16)  0.045; 0.101 | 1.03 ± 0.22  (0.93 – 1.12)  1.05 ± 0.16  (0.98 – 1.12)  1.03 ± 0.20  (0.94 – 1.12)  0.516; 0.030 | 1.03 ± 0.23  (0.91 – 1.14)  1.09 ± 0.29  (0.94 – 1.23)  1.01 ± 0.23  (0.89 – 1.13)  0.061; 0.152 | 1.03 ± 0.21  (0.93 – 1.13)  1.04 ± 0.21  (0.93 – 1.14)  1.04 ± 0.21  (0.94 – 1.14)  0.980; 0.001 |

The results are expressed as the mean ± standard deviation and 95% confidence interval (in parentheses). Data were analyzed with ANOVA with repeated measurements (RM ANOVA); the effect size expressed as partial eta square (*η*^2^*_p_*). Abbreviations: *BAS*, baseline; HMB, β-hydroxy-β-methylbutyrate; PLA, placebo; *SUP*, the first period of the only supplementation period during the usual training plan/lifestyle; *SUP+FGB*, the second period of the HMB/PLA treatment combined with the additional (in addition to their usual training plan/lifestyle) exercise stimuli in the form of two *Fight Gone Bad* (FGB) training units per week.
